# Constitutive *ALS3* expression in *Candida albicans* enhances adhesion and biofilm formation of *efg1*, but not *cph1* mutant strains

**DOI:** 10.1371/journal.pone.0286547

**Published:** 2023-07-13

**Authors:** Nicholas C. Schena, Kassandra M. Baker, Anna A. Stark, Derek P. Thomas, Ian A. Cleary

**Affiliations:** Department of Biomedical Sciences, Grand Valley State University, Allendale, Michigan, United States of America; University of Pennsylvania, UNITED STATES

## Abstract

Adhesion to living and non-living surfaces is an important virulence trait of the fungal pathogen *Candida albicans*. Biofilm formation in this organism depends on the expression of a number of cell surface proteins including the hypha-specific protein Als3p. Loss of *ALS3* impairs biofilm formation and decreases cell-cell adhesion. We wanted to test whether constitutively expressing *ALS3* could compensate for defects in adhesion and biofilm formation observed in mutant strains that lack key transcriptional regulators of biofilm formation Efg1p and Cph1p. We found that *ALS3* improved adhesion and biofilm formation in the *efg1*Δ and *efg1*Δ *cph1*Δ mutant strains, but had less effect on the *cph1*Δ strain.

## Introduction

*Candida albicans* is an opportunistic fungal pathogen that is a major threat to human health, particularly through its ability to cause disseminated, potentially fatal, infections in immune compromised patients [1]. A key virulence trait of *C*. *albicans* is biofilm formation on living and non-living surfaces. Biofilms on indwelling venous catheters can be a source and reservoir for disseminated infection [2]. *C*. *albicans* adhesion to surfaces and to other cells is mediated by surface proteins [3]. A number of genes expressed in a hypha-specific fashion encode surface proteins involved in adhesion, such as Als1p, Als3p, Hwp1 and Hwp 2 [4]. Als3p is a member of a family of agglutinin-like proteins in *C*. *albicans* and is found on hyphae [5]. Als3p has been implicated in a variety of virulence roles including binding to host cadherins [6], binding to ferritin [7] and biofilm formation [8], but is dispensable for virulence in a murine tail-vein injection model of disseminated candidiasis where biofilm formation may be less important [9]. Als3p also appears important for interactions with *Staphylococcus aureus* in polymicrobial infections [10, 11]. While loss of Als3p has a strong effect on biofilm formation on silicone elastomer, expression of other adhesins is not upregulated to compensate [9] and other members of the *ALS* family, while sharing some sequence similarity, have different patterns of expression [12] and appear to have specialized function [13].

Expression of these hyphal surface adhesins is stimulated in response to environmental changes that ultimately affect transcription factors such as Efg1p, Cph1p and Tec1p [4]. Mutants in such regulators can affect the morphological transition as well as expression of adhesins. *EFG1* encodes a transcription factor that is the focus of the cAMP pathway although it is also involved in responses to pH and N-acetyl glucosamine [4]. An *efg1*Δ mutant is unable to form hyphae in most inducing conditions [14] and is defective in biofilm formation [15], although it is hyperfilamentous in embedded conditions [16]. Interestingly, the *efg1*Δ mutant cells retain invasive characteristics in spite of their reduced adhesive properties [17].

Cph1p responds primarily to the MAPK pathway whose signals include serum and elevated temperature [4]. Loss of *CPH1* results in hypha formation defects in some conditions, such as on solid Spider and Lee media [18] and synthetic succinate [19], but does not impair hypha formation in others, like liquid Lee medium [18]. A double mutant lacking both of these genes has a stronger defect than either one individually and does not form hyphae in response to a range of external stimuli [20]. Although the double mutant is defective in biofilm formation on plastic [15, 21], it does retain some capacity for forming yeasty biofilms on glass [21].

In an *in vitro* catheter model, an *als3*Δ strain formed biofilms that comprised parallel rather than intertwined hyphae, adhered poorly to the surface, broke into numerous pieces when disturbed and had reduced mass. These defects were ameliorated when a copy of ALS3 was reintroduced [8]. We wanted to test whether constitutively expressing *ALS3* in strains lacking *EFG1* and/or *CPH1* could rescue adhesion or biofilm formation defects in various media. *ALS3* expression depends on *EFG1* through the transcription factors *TEC1* and *BCR1* [22–25] and to a lesser degree on *CPH1 [25]* so mutant strains in these genes have lower levels of Als3p than a wild-type strain. We found that constitutive *ALS3* expression had limited effects on the *cph1*Δ mutant strain but was able to improve adhesion and biofilm formation in the *efg1*Δ mutant and to a lesser degree the *efg1*Δ *cph1*Δ mutant strains.

## Materials and methods

### Strains and media

The yeast strains used in this study are listed in Table 1. Strains were routinely maintained as frozen -80°C stocks and grown on yeast extract-peptone-dextrose (YPD). All plasmid manipulations were performed with *Escherichia coli* strain DH5α with selection on Luria-Bertani plates containing 100 μg ml^−1^ ampicillin when necessary.

**Table 1:** *Candida albicans* strains used in this study.

Strain	Parent	Genotype^1^	Reference
Wild-type (SC5314)		Wild-type	[26]
*pACT1 ALS3*	SC5314	*RPS1*/*RPS1*::*SAT1- pACT1-ALS3*	This study.
*efg1*Δ (HLC52)		*iro1-ura3*Δ::*λimm* ^434^/*iro1-ura3*Δ::*λimm* ^434^ *efg1*Δ::*hisG/efg1*Δ::*hisG-URA3-hisG*	[20]
*efg1*Δ *pACT1 ALS3*	*efg1*Δ	*RPS1*/*RPS1*::*SAT1- pACT1-ALS3*	This study.
*efg1*Δ *cph1*Δ (HLC54)		*iro1-ura3*Δ:: λ *imm*^*434*^*/ iro1-ura3*Δ:: λ*imm*^*434*^ *cph1*::*hisG/cph1*::*hisG efg1*::*hisG/efg1*::*hisG-URA3-hisG*	[20]
*efg1*Δ *cph1*Δ *pACT1 ALS3*	*efg1*Δ *cph1*Δ	*RPS1*/*RPS1*::*SAT1- pACT1-ALS3*	This study
*cph1*Δ (JKC19)		*ura3*::*1 imm*^*434*^*/ura3*::*1 imm*^*434*^ *cph1*::*hisG/cph1*::*hisG-URA3-hisG*	[18]
*cph1*Δ *pACT1 ALS3*	*cph1*Δ	*RPS1*/*RPS1*::*SAT1- pACT1-ALS3*	This study
2322		*iro1-ura3*Δ:: λ *imm*^*434*^/*iro1-ura3*Δ:: λ *imm*^*434*^ *als3la*Δ/*als3sa*Δ-*ALS3LA-URA3*	[8]

^1^ The full genotype is that of the parental strain with additional modifications as indicated.

### Strain construction

The long allele of *ALS3* was PCR amplified in two overlapping sections from strain 2322 using primer pair ALS3_UPS2 5’-CTCGAGTATTAGATGCTACAACAATATACATT-3’ and ALS3_Nterm_Rev 5’-GAATTCAGTTGGGTTTGGCAGTGG-3’ with Expand^™^ High FidelityPLUS PCR System (Roche) and primer pair ALS3_Cterm_For_XhoI 5’-CTCGAGTACTAATCCAACTGACTCAATAGACAC-3’ and ALS3_Rev_EcoRI 5’-GAATTCAACATTTTCCTTGGACCTACTAC-3’ (modified from [8]) with Vent^®^ DNA Polymerase (New England Biolabs). The PCR products were cloned into plasmid pMiniT using the NEB PCR Cloning Kit (New England Biolabs) and the sequence analyzed to confirm that there were no PCR errors. The two pieces were sequentially cloned into plasmid CIpSATSA [27] using the restriction sites in the primers and an internal *BSP*14071 site in the overlapping region of the two fragments. The resulting plasmid has the full-length coding sequence of *ALS3* under the control of the *ACT1* promoter sequence.

To construct constitutive *ALS3* expression strains, *Candida albicans* strains were transformed using a modified electroporation transformation method [28] using linearized plasmid CIpSATSAALS3 to integrate a constitutively-expressed copy of *ALS3* at an ectopic site. Nourseothricin-resistant transformants were selected on YPD agar plates containing 200 μg ml^–1^ nourseothricin (Werner Bioagents) as described previously [29]. Correct integration of the construct was confirmed by PCR.

### Slide adhesion

*C*. *albicans* cells from overnight cultures were washed in sterile phosphate buffered saline (PBS), counted and then resuspended at a concentration of 2x10^8^ CFU/ml in RPMI-1640. 10μl of each strain was placed on a glass microscope slide and the slide placed in a hybridization chamber with water added to the reservoirs to maintain humidity. The chambers were then incubated at 37°C for 3h. After incubation the slides were removed and photographed. The slides were then gently washed with 1ml of sterile water and photographed again. Assays were done in biological triplicate.

### Morphology assays

Strains were grown overnight shaking in YPD 28°C and diluted 1/20 into fresh medium. (RPMI-1640 buffered with MOPS, Spider [18] or GlcNAc [30]) and grown shaking for at 37ºC for 4h. Cells were then removed and photographed. Assays were done in biological triplicate. For each replicate, 8–10 fields of view were photographed, and the cultures and micrographs were analyzed independently by at least two of the authors.

### Biofilm formation

Biofilm formation was assessed using the 96-well plate model described in [31]. Briefly, Cells from an overnight culture were washed, counted and resuspended to a final concentration of 1x10^6^ CFU/mL in RPMI-1640, GlcNAc or Spider medium. Aliquots of 100μl were used to seed wells in the 96-well plate. Plates were incubated for 24h at 37°C, washed to remove non-adherent cells and stained with crystal violet. Biofilms were washed with sterile water to remove excess stain, then destained with 33% acetic acid [32]. The supernatant was transferred to empty wells and the OD_550_ measured using a plate reader (BioTek). The results were analyzed by Student’s t-test, comparing each transformant strain with its parent strain.

## Results

### Adhesion to glass slides

**Fig 1:**
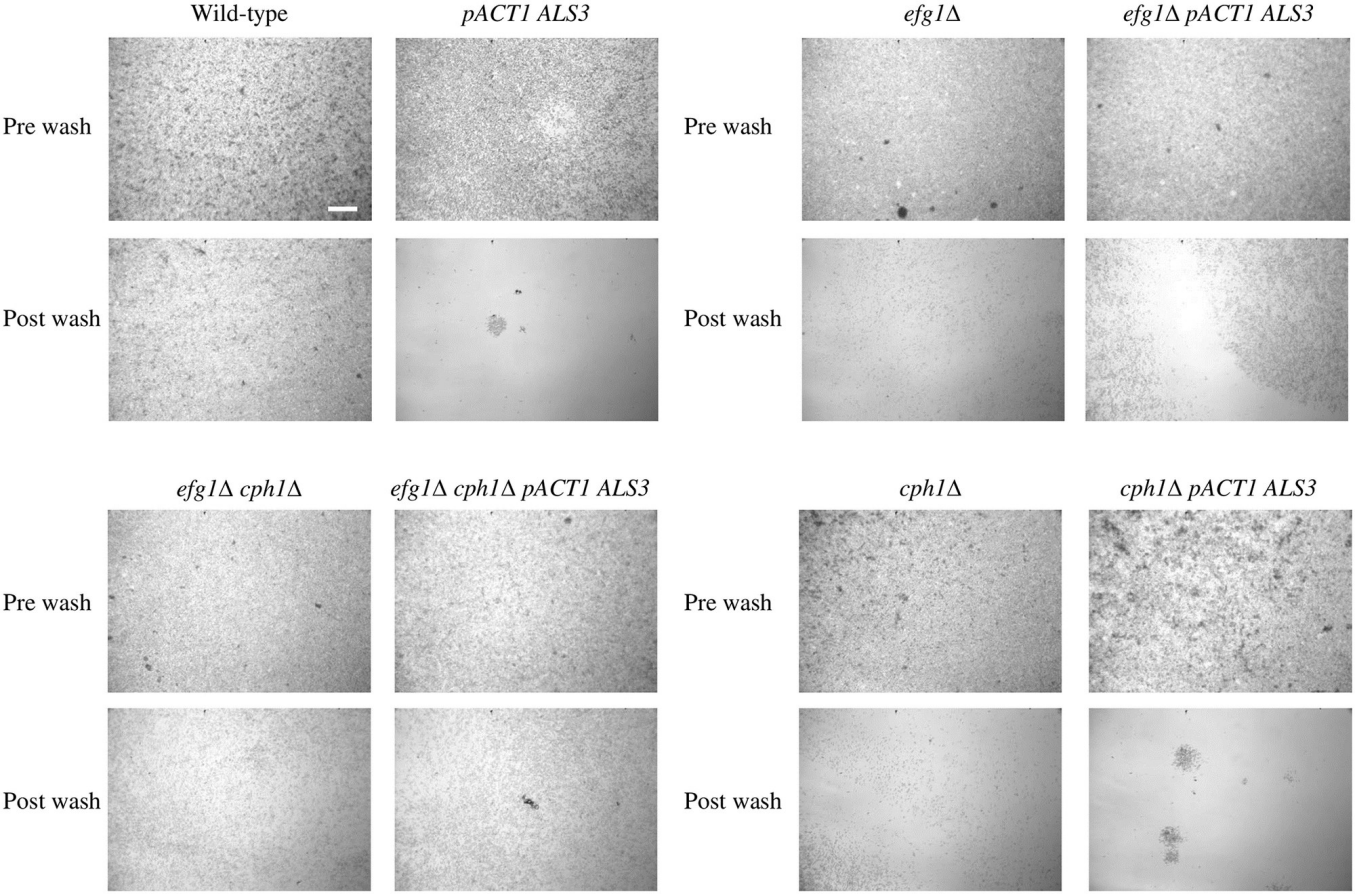
Slide adhesion. Strains were spotted onto glass slides and incubated at 37ºC for 3h. Cells were photographed before (pre wash) and after (post wash) gentle washing with 1ml sterile water. Constitutive *ALS3* expression in a wild-type strain resulted in fewer cells remaining attached after washing, whereas constitutive *ALS3* expression in the *efg1*Δ mutant resulted in more cells remaining. There was little difference in the *efg1*Δ *cph1*Δ mutant with or without constitutive *ALS3* expression, and whilst most cells of the *cph1*Δ washed off even with constitutive *ALS3* expression, the remaining cells were clumped rather than evenly dispersed. The scale bar represents 50 microns.

Changes in cell surface proteins can affect adhesion to cells and to solid surfaces. To examine surface adhesion, we placed cells on glass slides and examined them before and after washing (Fig 1). In the wild-type, washing removed some cells, but most remained on the slide. Surprisingly, constitutive *ALS3* expression in this background resulted in more cells washing off the slide. Washing removed nearly all the *efg1*Δ mutant cells while more remained in the *ALS3* constitutive expression strain. In the *efg1*Δ *cph1*Δ mutant many cells remained after washing and constitutive *ALS3* expression did not alter that result. With the *cph1*Δ mutant, most cells washed away and constitutive *ALS3* expression resulted in a few dense clumps remaining with the bulk of cells still washing away. This was most similar to the wild-type, although more of the mutant cells washed away.

### Growth in liquid media

**Fig 2:**
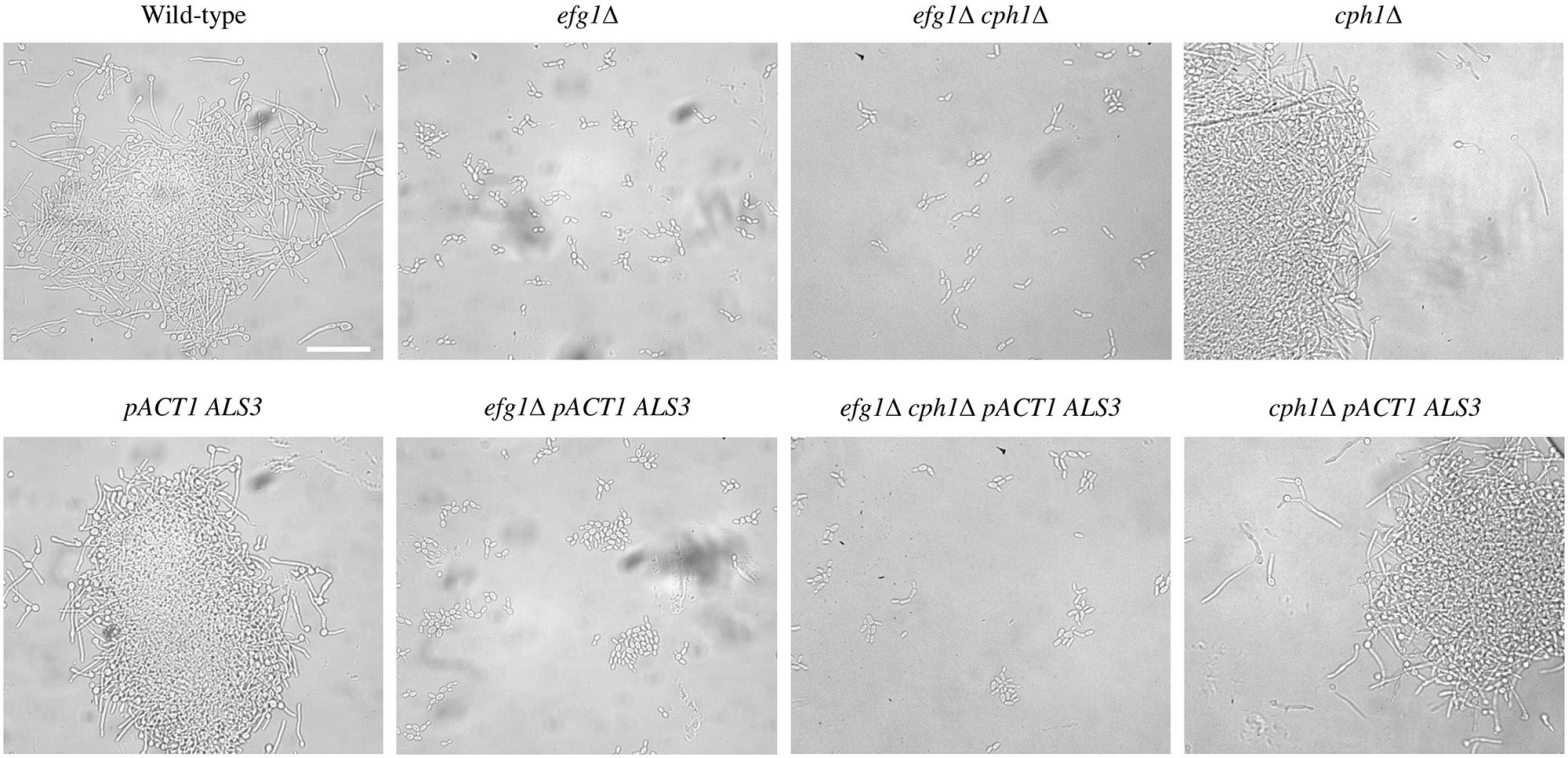
Liquid cultures in RPMI-1640. Strains were grown overnight at 28ºC then diluted 1/20 in RPMI-1640 and grown shaking at 37ºC for 4h. Constitutive *ALS3* expression resulted in greater cell clumping in the *efg1*Δ mutant, a small increase in the *efg1*Δ *cph1*Δ strain and no substantial effect on cell adhesion in other two strains. The scale bar represents 50 microns.

**Fig 3:**
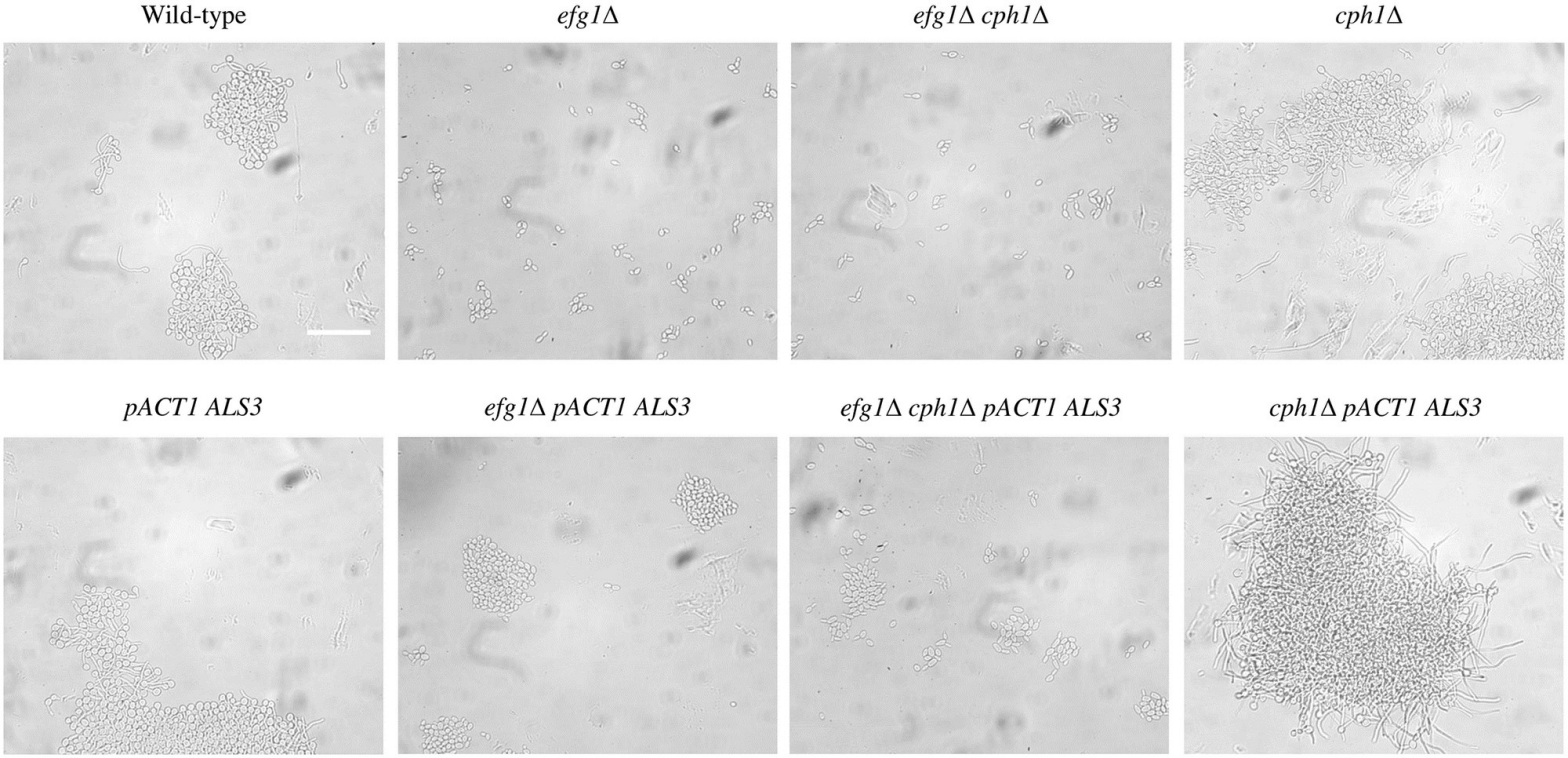
Liquid cultures in Spider. Strains were grown overnight at 28ºC then diluted 1/20 in Spider medium and grown shaking at 37ºC for 4h. Constitutive *ALS3* expression resulted in greater cell clumping in the *efg1*Δ and *efg1*Δ *cph1*Δ backgrounds, but did not have a substantial effect on cell adhesion in other strains. The scale bar represents 50 microns.

**Fig 4:**
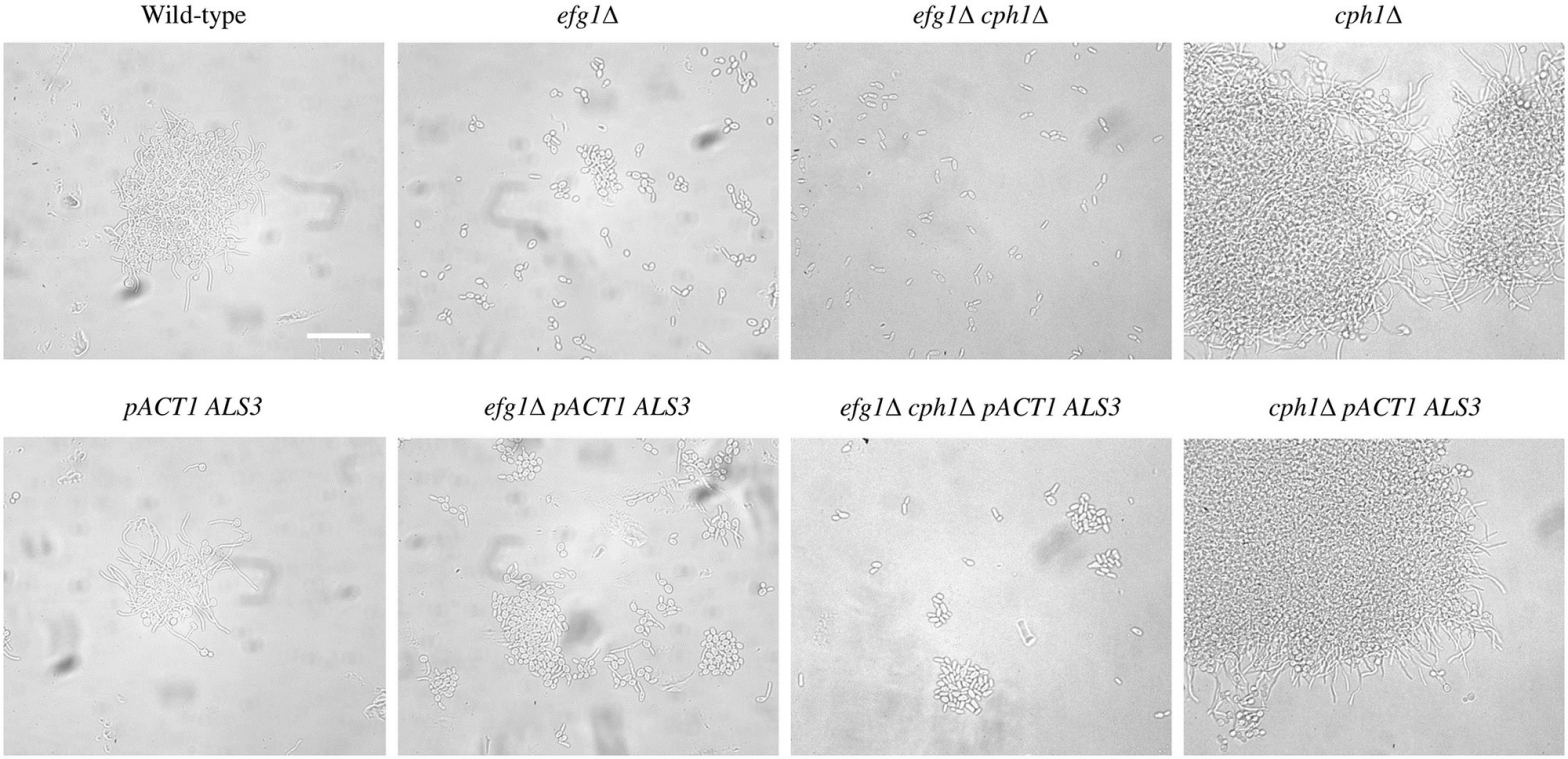
Liquid cultures in GlcNAc. Strains were grown overnight at 28ºC then diluted 1/20 in GlcNAc medium and grown shaking at 37ºC for 4h. Constitutive *ALS3* expression resulted in greater cell clumping in the three mutant strains, but not the wild-type. The scale bar represents 50 microns.

To test whether constitutive *ALS3* expression could affect adhesion between cells in liquid media, strains were grown shaking in RPMI-1640, Spider and GlcNAc at 37ºC for 4 hours. In all three of these media, the wild-type and the *cph1*Δ strains formed hyphae, whereas the *efg1*Δ and *efg1*Δ *cph1*Δ mutants grew as individual or short chains of elongated yeast cells (Fig 2). Constitutive expression of *ALS3* did not alter the morphology of any of these strains. In RPMI-1640, Cells of the wild-type and *cph1*Δ strains formed large clumps with and without constitutive *ALS3* expression. In the *efg1*Δ background, constitutive *ALS3* expression resulted in the formation of numerous small clumps of cells compared to the parent strain where most cells were single or in short chains. A similar but less pronounced change was seen in the *efg1*Δ *cph1*Δ strain with constitutive *ALS3* expression. In Spider medium a similar pattern to RPMI-1640 was seen with constitutive *ALS3* expression not affecting cell clumping in wild-type or *cph1*Δ backgrounds, but increased clumping in the *efg1*Δ and *efg1*Δ *cph1*Δ mutant backgrounds (Fig 3). In GlcNAc medium, constitutive *ALS3* expression resulted in increased clumping in all three mutant backgrounds, but not in the wild-type background (Fig 4).

### Biofilm formation

**Fig 5:**
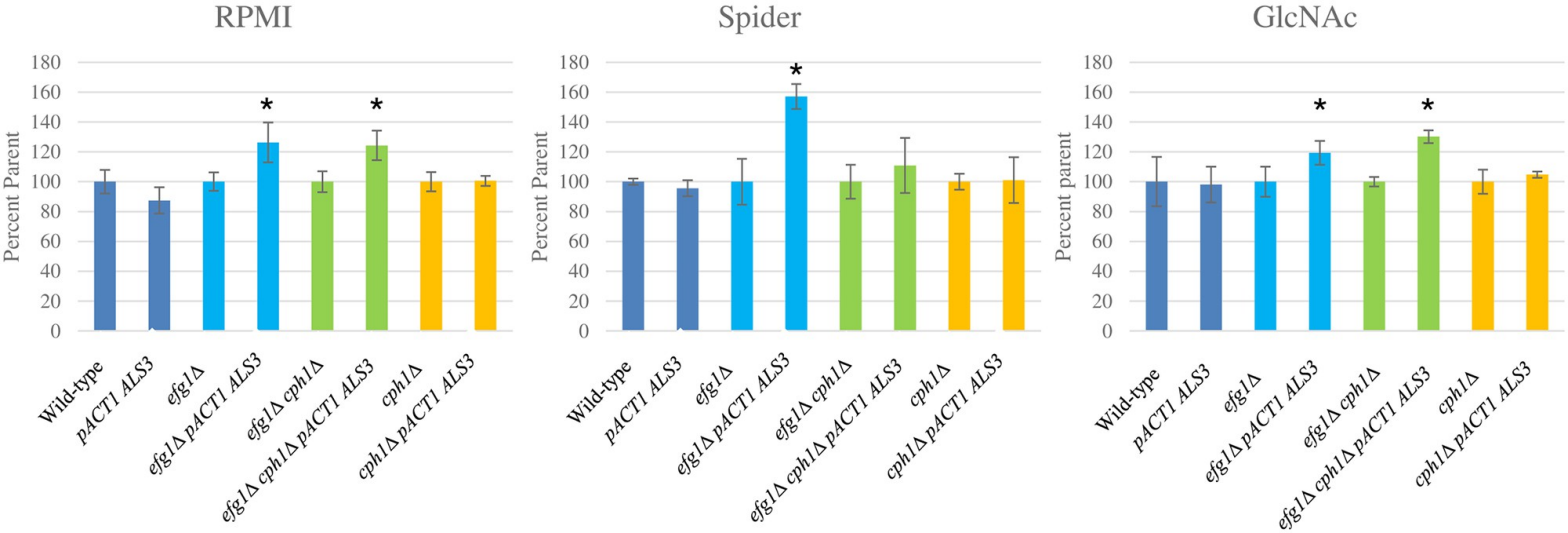
Biofilm formation. Biofilm formation was assessed using the 96-well plate model and results were quantified by crystal violet staining. Biofilm formation is expressed as percentage of the parent strain, which is set to 100%, and the pairs of strains are indicated by colour. Dark blue, wild-type; Light blue, *efg1*Δ; Green *efg1*Δ *cph1*Δ; Yellow, *cph1*Δ. Asterisks indicate statistically significant differences between the transformant and the parent strain. In RPMI-1640, constitutive *ALS3* expression increased biofilm formation in the *efg1*Δ and *efg1*Δ *cph1*Δ strains (P<0.05). In Spider, constitutive *ALS3* expression increased biofilm formation in the *efg1*Δ strain (P<0.05). In GlcNAc constitutive *ALS3* expression increased biofilm formation in the *efg1*Δ and *efg1*Δ *cph1*Δ strains (P<0.05).

Biofilm formation was assessed in RPMI-1640, Spider and GlcNAc media using a 96-well plate model with crystal violet staining. The results are expressed as biofilm formation of the constitutive expression strain as a percentage of its parent, thus the wild-type and each mutant strain is expressed as 100% (Fig 5). In RPMI-1640 constitutive *ALS3* expression resulted in statistically significant increases in biofilm formation in the *efg1*Δ and *efg1*Δ *cph1*Δ mutant strains but no statistically significant changes in biofilm formation in the wild-type or in the *cph1*Δ mutant. In Spider medium constitutive *ALS3* expression resulted in a statistically significant increase in biofilm formation in the *efg1*Δ background but biofilm formation was essentially unchanged in the other strains. In GlcNAc medium constitutive *ALS3* expression resulted in statistically significantly increased biofilm formation in the *efg1*Δ and *efg1*Δ *cph1*Δ mutant strains but did not affect biofilm formation in the wild-type or the *cph1*Δ mutant strains (Fig 5).

## Discussion

Adhesion is important for many aspects of *C*. *albicans* biology. Yeast cells adhere best to vascular cells under flow conditions [33], an important aspect of escaping the bloodstream during infection. Biofilm formation is important for growing on implanted medical devices and causing disease, and also for mating [34]. The transition from yeast to hyphae in *C*. *albicans* is associated with numerous changes in cell wall composition that are important for adhesion to other cells and to non-living surfaces in the establishment of biofilms. Strains locked in the yeast form have substantially reduced biofilm forming ability [15, 35] as do mutants in surface adhesins like Als3p [8]. This therefore led us to ask whether constitutive expression of a single one of these surface proteins, *ALS3*, could rescue adhesion and biofilm defects in *efg1*Δ and *cph1*Δ mutant strains, which have reduced *ALS3* expression [22–25].

Although *ALS3* is normally expressed in hyphae and is abundant in a wild-type strain, we also tested the effects of constitutive expression in this situation. In general, this did not affect hypha formation, cell adhesion in liquid media or biofilm formation. Interestingly, more cells of the constitutive expression strain washed off the glass slides than the wild-type. Perhaps the additional Als3p increased the adhesion between the *C*. *albicans* cells so that they are more easily removed *en masse* from the surface. Alternatively, since different members of the Als family have different temporal and physical distributions [36], perhaps constitutive Als3p production disrupts the normal distribution of cell wall proteins in a way that actually causes a decrease in adhesion. Indeed, it has been suggested that perturbations of the normal hyphal cell wall adhesin complement could disrupt the typical phenotype of these cells [13], and perhaps in our strain additional Als3p facilitates the protein binding more to itself than to the glass or perhaps adhesins that typically interact with the glass are being obscured by the additional Als3p.

The *efg1*Δ mutant does not form hyphae in any of the conditions we tested. The cells were easily washed off the glass slide, but constitutive *ALS3* expression resulted in more cells remaining on the surface. Constitutive *ALS3* expression did not alter the cellular morphology but did increase cell clumping in the three liquid media tested and resulted in increased biofilm formation in the same media, particularly in spider.

The *efg1*Δ *cph1*Δ double mutant has a strong defect in hypha formation [20] and biofilm formation [15], although it retains some ability to adhere to glass [21]. In our assay more cells of the double mutant strain remained attached to the glass slide after washing than the two single deletion strains, although more cells washed off than the wild-type strain. Constitutive *ALS3* expression did not alter this phenotype, in contrast to the *efg1*Δ strain where *ALS3* expression did result in better adhesion. In all three liquid media tested no hyphae were formed but constitutive *ALS3* expression resulted in increased cell clumping. Constitutive *ALS3* expression also affected biofilm formation in this mutant background, with increased biofilm formation in RPMI and GlcNAc.

Of the three deletion strains examined here, the *cph1*Δ mutant strain had the least substantial defect in hypha formation in the conditions we tested and it is able to form hyphae in liquid media [20] and form biofilms [15]. It was, however, easily washed off glass slides. Constitutive expression of *ALS3* in the *cph1*Δ strain did not stop the cells from being washed away, but did cause the cells to clump more, and those clumps did remain on the glass. The adherent clumped cells resembled the result seen in the wild-type background with constitutive *ALS3* expression. In liquid culture constitutive *ALS3* expression did not alter hypha formation but did appear to increase clumping in GlcNAc. Despite the changes seen on slides and in liquid culture, biofilm formation was the same with or without constitutive *ALS3* expression.

Where constitutive *ALS3* expression has affected adhesion and biofilm formation in these mutant strains it has nonetheless not produced a wild-type phenotype. A number of surface proteins are upregulated in normal hypha formation so it is perhaps to be expected that increasing the production of one would not be sufficient to restore normal properties. However, constitutively expressing this single gene did improve adhesion and biofilm formation in strains with known defects, notably in strains missing *EFG1* that are unable to filament in the conditions we tested.
